# Supplementary material for: Atomic structures and orbital energies of 61,489 crystal-forming organic molecules
Source: Sci Data. 2020 Feb 18;7:58. doi: 10.1038/s41597-020-0385-y (PMC7029047; doi:10.1038/s41597-020-0385-y)
Supplement: Supplementary file 1 — Supplementary Information [file 41597_2020_385_MOESM1_ESM.pdf]

Supplementary Information

Contents

1) Computational settings
2) def2-QZVP basis set for iodine
3) Technical validation of all occupied G_0W_0 quasiparticle states

1) Computational settings

DFT PBE+vdW (vacuum) calculations

```
#General settings:
xc                          pbe
spin                        none
relativistic                atomic_zora scalar
occupation_type             gaussian 0.01
mixer                       pulay
n_max_pulay                 10
charge                      0
charge_mix_param            0.2
default_initial_moment      0

# Convergence criteria:
sc_accuracy_eev             1E-3
sc_accuracy_etot            1E-6
sc_accuracy_rho             1E-5
sc_iter_limit               100

# Other settings:
prodbas_threshold           1.e-4
relax_geometry              trm 1E-3
vdw_correction_hirshfeld
packed_matrix_format        index
```

DFT PBE0 (vacuum) calculations

```
#General settings:
xc                                 pbe0
spin                               none
relativistic                       atomic_zora scalar
occupation_type                    gaussian 0.01
mixer                              pulay
n_max_pulay                        10
charge                             0
charge_mix_param                   0.2
default_initial_moment             0
```

```
# Convergence criteria:
sc_accuracy_rho                    1E-5
sc_accuracy_eev                    1E-3
sc_accuracy_etot                   1E-6
sc_iter_limit                      100

# Other settings:
prodbas_threshold                  1.e-4
vdw_correction_hirshfeld
packed_matrix_format               index
```

DFT PBE0 (water) calculations

```
#General settings:
xc                                 pbe0
spin                               none
relativistic                       atomic_zora scalar
occupation_type                    gaussian 0.01
mixer                              pulay
n_max_pulay                        10
charge                             0
charge_mix_param                   0.2
default_initial_moment             0


# Convergence criteria:
sc_accuracy_rho                    1E-5
sc_accuracy_eev                    1E-3
sc_accuracy_etot                   1E-6
sc_iter_limit                      100

# Other settings:
prodbas_threshold                  1.e-4
vdw_correction_hirshfeld
packed_matrix_format               index
solvent                            mpe
mpe_solvent_permittivity           78.36
isc_cavity_type                    rho_free 0.0121
mpe_nonelectrostatic_model         linear_OV 0.00291 -0.00225
mpe_lmax_rf                        14.0
mpe_degree_of_determination        16.0
mpe_lmax_ep                        8.0
isc_max_dyn_steps                  1000
isc_kill_ratio                     1.0e-3
```

G_0W_0@PBE0 calculations

```
#General settings:
qpe_calc           gw
xc                 pbe0
relativistic       atomic_zora scalar
occupation_type     gaussian 0.0001
mixer               pulay
```

```
n_max_pulay        10
charge_mix_param   0.2

# Convergence criteria:
sc_accuracy_rho    1E-6
sc_accuracy_eev    1E-4
sc_accuracy_etot   1E-6
sc_iter_limit      100

# Other settings:
prodbas_threshold  1.e-4
anacon_type       1
frequency_points  200
n_anacon_par      16
override_illconditioning .true.
RI_method         v
```

2) def2-QZVP basis set for iodine

```
species          I
# global species definitions
nucleus        53
mass           126.90447
l_hartree           6
cut_pot        4.0  2.0  1.0
basis_dep_cutoff    0.d0
radial_base    64  7.0
radial_multiplier  6
angular_grids       specified
 division   1.1798  194
 division   1.3657  302
 division   1.5819  434
 division   1.7978  590
 outer_grid  770
```

```
# Definition of "minimal" basis
# valence basis states
    valence      5  s   2.
    valence      5  p   5.
    valence      4  d  10.
# ion occupancy
    ion_occ      5  s   1.
    ion_occ      5  p   4.
    ion_occ      4  d  10.
```

```
#   For exact comparison with all GTO-based codes, one or more of
#   the following flags are needed:

    include_min_basis   .false.
    pure_gauss          .true.
```

```
#I   def2-QZVP

gaussian 0 12
  2858800923.1      0.93180377998E-08
  362947182.93      0.29251660320E-06
  25895129.209      0.49238597444E-05
  4460334.5360      0.38960258418E-04
  880899.58248      0.25876591258E-03
  221418.55804      0.12385841119E-02
  66902.706606      0.48235267455E-02
  23226.795213      0.15583064855E-01
  8615.8529208      0.46441583562E-01
  3573.9145324      0.87051184822E-01
  1794.5824834      0.11343892900
  850.45867247      0.54927921531E-01
gaussian 0 4
  14239.560861      0.52195064531E-02
  3820.1734359      0.73018805288E-01
  1448.8542868      0.35799348636
  762.11441290      0.76902430693
gaussian 0 1  400.07563376
gaussian 0 1  207.99586804
gaussian 0 1  93.207388154
gaussian 0 1  47.765459426
gaussian 0 1  24.550232786
gaussian 0 1  12.090879108
gaussian 0 1  6.2765010441
gaussian 0 1  2.7542647749
gaussian 0 1  1.3387135879
gaussian 0 1  0.49497418409
gaussian 0 1  0.25248622219
gaussian 0 1  0.10298927907
gaussian 1 9
  81817.656222      0.39698900367E-04
  19362.496816      0.35499624328E-03
  6270.1866595      0.20713055193E-02
  2392.8261774      0.93203815875E-02
  1013.4368905      0.33817222320E-01
  464.22760436      0.97537230922E-01
  221.75579791      0.22849921788
  109.80480104      0.37589451352
  55.045742477      0.33085898686
gaussian 1 5
  3490.9946288      0.88809282998E-04
  449.22593362      0.16224273869E-02
  121.43614113     -0.20946984795E-01
  26.657569747      0.18421783179
  9.8528237930      0.29164798721E-01
gaussian 1 4
  351.70093301     -0.76407514730E-02
  56.155281403     -0.10510879882E-02
  13.455175872      0.61521964431
  6.5609918621      0.36020414662
gaussian 1 2
  4.2887611403      0.35087173331
  2.2526244212      0.65636775384
```

```
gaussian 1 1  1.1534418891
gaussian 1 1 0.48252007546
gaussian 1 1 0.20049713189
gaussian 1 1 0.77422160270E-01
gaussian 2 8
  5202.7564499      0.55860101511E-04
  1561.5422849      0.58346576529E-03
  594.48186578      0.39438604353E-02
  252.58534246      0.20100542180E-01
  114.00698111      0.76566629696E-01
  53.528733479      0.21093133804
  25.720582750      0.37509517643
  12.496559121      0.36939862363
gaussian 2 3
  51.415770527     -0.44511873536E-02
  12.994996871      0.46399746597E-01
  6.0907370410      0.39721573389
gaussian 2 1  2.8294142727
gaussian 2 1  1.2670737878
gaussian 2 1  0.51177746489
gaussian 2 1  0.21000000000
gaussian 3 1  0.58650000000
gaussian 3 1  0.24940000000
gaussian 3 1  2.1780000000
gaussian 3 1  6.4253613600
gaussian 4 1  0.47300000000
```

3) Technical validation of all occupied G_0W_0 quasiparticle states

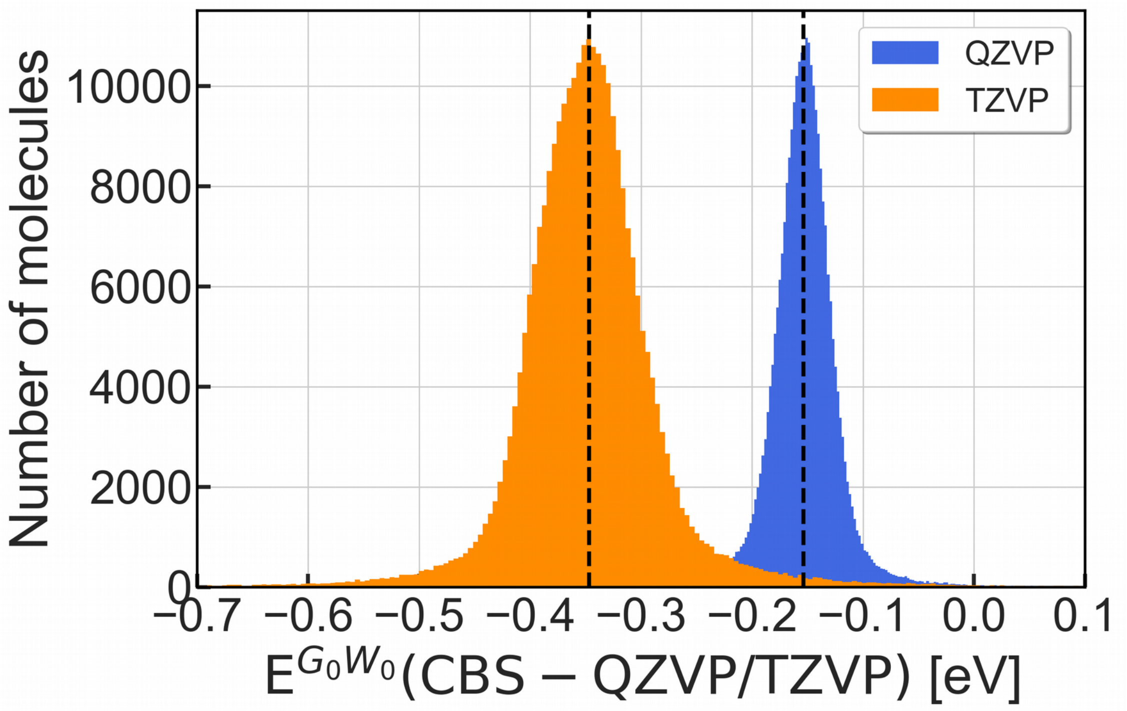

Figure 1: Deviation of all occupied states from the CBS limit for the 5k subset. The distributions are centered around -0.35 eV (TZVP) and -0.15 eV (QZVP), with a standard deviation of 0.03 eV (TZVP) and 0.02 (QZVP).
